# Foam Stability in Aqueous Systems Containing an Amino Acid-Based Surfactant and Gelatin: An Interfacial Shear Rheology Perspective

**DOI:** 10.1021/acs.langmuir.5c05853

**Published:** 2026-04-10

**Authors:** Kenta Asai, Kyosuke Arakawa, Koji Tsuchiya, Shiho Yada, Yukishige Kondo, Yoshifumi Yamagata, Hideki Sakai, Kenichi Sakai

**Affiliations:** † Department of Pure and Applied Chemistry, Faculty of Science and Technology, 26413 Tokyo University of Science, 2641 Yamazaki, Noda, Chiba 278-8510, Japan; ‡ Research Institute for Science and Technology, Tokyo University of Science, 2641 Yamazaki, Noda, Chiba 278-8510, Japan; § Department of Industrial Chemistry, Faculty of Engineering, Tokyo University of Science, 6-3-1 Niijyuku, Katsushika, Tokyo 125-8585, Japan; ∥ Anton Paar Japan K.K. Riverside Sumida 1F, 1-19-9 Tsutsumi-dori, Sumida, Tokyo 131-0034, Japan

## Abstract

Interfacial shear rheological measurements using a bicone-type geometry were conducted to investigate the role of interfacial shear properties in foam stability. Two materials were employed as foam stabilizers: an anionic amino acid-based surfactant and a zwitterionic polyelectrolyte (gelatin). The rheological analysis indicated that their mixture forms a viscoelastic film adsorbed at the air/solution interface. At a surfactant concentration of 5 mmol/dm^3^ in the presence of gelatin (0.5 mass %) at pH 7, both the interfacial shear storage modulus (*G*′) and loss modulus (*G*″) reached maximum values, while the loss tangent (tan δ = *G*″/*G*′) reached a minimum, indicating the formation of an elasticity-dominant, gel-like interfacial film. These results suggest that interfacial gelation occurs between the oppositely charged components. Notably, the highest foam stability was observed at the composition, implying that interfacial gelation enhances foam stability by suppressing liquid drainage and bubble coalescence. Furthermore, interfacial rheological measurements showed that lower pH promotes more pronounced elastic responses, consistent with lower surface tension and improved foam stability. These findings highlight the potential of interfacial shear rheology using the bicone-type geometry as a practical tool for predicting the long-term stability of foams in various industrial applications.

## Graphical abstract

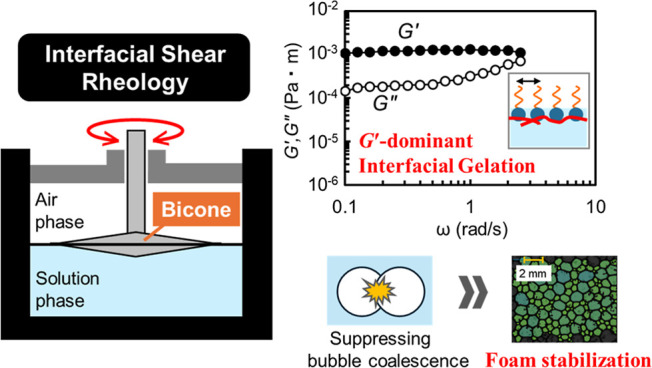

## Introduction

Foams are nonequilibrium colloidal dispersion systems composed of a dispersed gas phase and a continuous liquid phase. They undergo destabilization through several mechanisms, including gas diffusion between bubbles (Ostwald ripening), drainage from the liquid film between two air/liquid interfaces, and bubble coalescence.^1^ Disjoining pressure, defined as interaction forces between two opposing air/liquid interfaces per unit area, also plays a significant role in foam stability.^2^ These interaction forces are affected not only by the composition of molecular films formed at the air/liquid interface but also by the structural organization of fluids within the liquid film.^2^

These destabilization processes are expected to be influenced by the mechanical or rheological properties of molecular films formed at the air/liquid interface. The most common method for characterizing these rheological properties is interfacial dilational rheology, which monitors changes in surface tension during the expansion and compression of air/liquid interfaces, typically using a liquid droplet in air or a bubble formed in the liquid. This technique was reviewed by Ravera et al.,^3^ and additional surfactant systems have been studied since then.^4–6^ The stability of foams has also been discussed based on dilational rheological data.^7–14^ Some studies have suggested that interfacial dilational elasticity is a key parameter for predicting foam stability. For example, the addition of an anionic surfactant to an aqueous solution of a cationic gemini surfactant increased the packing density of surfactant molecules adsorbed at the air/solution interface, resulting in increased interfacial dilational elasticity of the mixed film.^10^ This change contributed to increased foam stability.^10^ In aqueous mixtures of a cationic surfactant and an anionic polyelectrolyte, the interfacial dilational elasticity was maximized near their critical aggregation concentration, where the highest foam stability was also observed.^13^

Interfacial shear rheology measurements using a bicone-type geometry^15–18^ are also useful for assessing rheological properties at air/liquid interfaces^19–22^ as well as liquid/liquid interfaces.^23–25^ This method can directly evaluate the two-dimensional mechanical properties of interfaces under applied shear flow. It can isolate the rheological responses of an interfacial film from those of the bulk liquid phase, providing insights into interfacial phenomena that cannot be obtained from conventional bulk rheology measurements.

At the air/water interface, the film can be formed either through spontaneous adsorption of film-forming materials from the aqueous phase or spreading of volatile oil droplets in which the materials are dissolved.^20^ The following examples highlight notable findings obtained using the former method. Espinosa and Langevin^19^ demonstrated that interfacial shear rheology depends on the molecular flexibility of polyelectrolytes in surfactant–polymer mixtures; “rigid” polyelectrolytes yield brittle interfacial films, whereas “more flexible” ones yield more viscoelastic or purely viscous films in aqueous mixtures of surfactants and polymers. Regarding foam stability, it has been reported that the combined use of 1-tetradecanoic acid and hexadecyltrimethylammonium chloride produces highly stable foams that resist both Ostwald ripening and bubble coalescence.^21,22^ This enhanced stability is attributed to the formation of a thick interfacial film comprising two distinct layers: (i) a highly viscoelastic monolayer adsorbed at the air/water interface and (ii) underlying elastic, gel-like multilayers formed by intact or fused vesicles with micrometer-scale thickness.^21,22^ However, studies investigating foam stability through interfacial shear rheology remain still limited, particularly those that use the bicone-type geometry.

In this study, we investigated the interfacial shear rheology of aqueous mixtures composed of an anionic amino acid-based surfactant (*N*-dodecanoylglutamic acid) and a zwitterionic polyelectrolyte (gelatin) to elucidate the role of interfacial shear properties in foam stability. Gelatin and anionic surfactants are known to form complexes not only in bulk solutions but also at the air/solution interface.^26^ The surfactant–gelatin complex is expected to form a thick interfacial film with pronounced viscoelastic properties and increase turbidity in the liquid phase under specific mixing ratios and pH conditions. These features make dilational rheology measurements particularly challenging. In contrast, interfacial shear rheology measurements using the bicone-type geometry provide a more reliable approach for characterizing the two-dimensional viscoelasticity of such complex interfaces.

## Materials and Methods

### Materials

*N*-Dodecanoylglutamic acid was synthesized via the Schotten–Baumann reaction of monosodium glutamate with dodecanoyl chloride. Acid-treated gelatin (Type A, from porcine skin) was purchased from Sigma-Aldrich (G2500, Lot. 0000250565) and used as received. HCl and NaOH were purchased from FUJIFILM Wako Pure Chemical Corporation. The pH of each sample solution was adjusted using a small amount of dilute aqueous HCl or NaOH. The water used in this study was purified using either a Millipore Direct-Q UV3 or a Millipore Milli-Q Reference water purification system. The water supplied by these systems was of “ultrapure” quality, with a displayed specific resistivity of 18.2 MΩ·cm.

### Methods

Static surface tension measurements were performed using a Krüss K100 Wilhelmy auto surface tensiometer with a platinum plate. Continuous measurements were conducted until the change in surface tension was less than 0.1 mN/m per 900 s. Three repeated measurements were performed for each data point.

Foamability and foam-stability were examined visually in accordance with the following procedure. Each sample (3 cm^3^) was poured into a cylindrical glass tube (diameter = 18 mm, height = 180 mm), and it was capped with a glass stopper. Then the glass tube was vigorously shaken by hands 20× (approximately 5 s). Foam stability was estimated as the ratio of the foam height measured 24 h after hand-shaking to that measured immediately after hand-shaking. Three repeated measurements were performed to estimate foam stability.

The morphology of foams was assessed using a Krüss DFA100 dynamic foam analyzer. Air was injected from the bottom of a cylindrical column until the foam height reached 15 cm from the bottom. The airflow was then stopped, and the morphology of the foams formed by bubbling was continuously monitored using a CCD camera positioned approximately 7 cm above the bottom.

Interfacial shear rheology was measured using an Anton Paar MCR302 rheometer equipped with a bicone-type geometry (BiC 68–2 × 5, disk radius = 34.14 mm, cone angle = 5°, cone penetration depth = 2.205 mm), a glass measuring cup (inner radius = 40.00 mm, height = 45.00 mm), and a heating/cooling jacket. The setup of this instrument was described in our recent study,^24^ although that work focused on an oil/water interface rather than the air/solution interface. Briefly, a sample solution was poured into the glass measuring cup, and the bicone-type geometry was positioned at the air/solution interface. Frequency-sweep measurements were performed at least 15 min after the setting under a constant strain of 1%. These measurements were performed repeatedly at least 3×.

Interfacial storage modulus *G*′ (Pa·m) and interfacial loss modulus *G*″ (Pa·m) were calculated by solving the Navier–Stokes equations for the bulk phase velocity field with the Boussinesq–Scriven boundary condition, which describes interfacial stress and strain by accounting for the coupling between the interface and the bulk phase. This calculation was performed using a program implementing the iterative scheme developed by Sánchez-Puga et al.^27^

In the measurements mentioned above, the surfactant concentration was varied from 0.1 to 50 mmol/dm^3^ in the presence of gelatin at a fixed concentration of 0.5 mass %. All measurements reported herein were performed at 25 °C.

## Results and Discussion

### Surface Tension

Static surface tension measurements were performed at different surfactant concentrations with and without gelatin (0.5 mass %). The solution pH was adjusted to 7, 9.5, or 11 using a small amount of HCl or NaOH. Zeta potential measurements confirmed that the isoelectric point of the acid-treated gelatin was approximately 9.5 (Figure S1). Therefore, gelatin carries a net positive charge at pH 7 and a net negative charge at pH 11, while the surfactant is negatively charged under all conditions.

Figure 1 shows the surface tension results. In the single-surfactant system (pH 7), the surface tension gradually decreased with increasing concentration and reached a plateau at approximately 14 mmol/dm^3^ (critical micelle concentration). In contrast, the surface tension in the mixed surfactant–gelatin system differed from that in the single-surfactant system even at the same pH. That is, the surface tension in the mixed surfactant–gelatin system decreased stepwise, exhibiting at least two or three plateau regions, irrespective of pH. Two-step decrease in surface tension is frequently observed in aqueous surfactant–polymer mixtures.^28,29^ As mentioned earlier, gelatin and anionic surfactants are expected to form complexes not only in bulk solutions but also at the air/solution interface.^26^ Considering the results reported in these references, the stepwise decrease in surface tension observed in Figure 1 can be reasonably interpreted as the adsorption of the surfactant–gelatin complex at the air/solution interface. The presence of one or two plateau regions at intermediate surfactant concentrations may also suggest a conformational rearrangement of the adsorbed complex. The final plateau (above 20 mmol/dm^3^) indicates the formation of a surfactant monolayer, resulting from the replacement of gelatin molecules adsorbed at the interface by surfactant molecules from the bulk solution.

**Figure 1:**
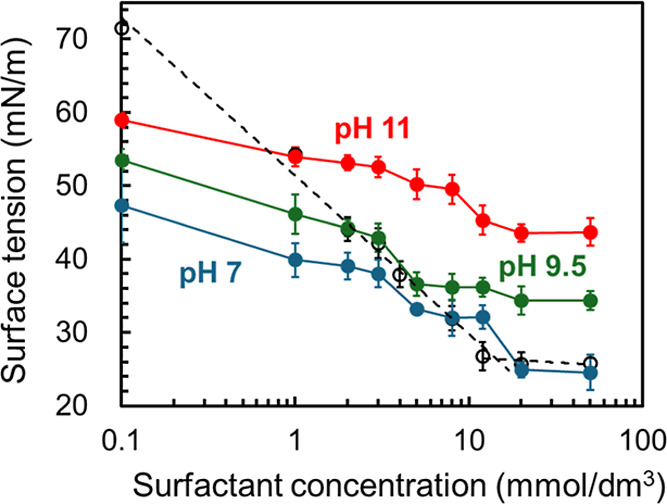
Surface tension of mixed surfactant–gelatin solutions as a function of surfactant concentration. The gelatin concentration was fixed at 0.5 mass %. The solution pH was adjusted to approximately 7 (solid blue circles), 9.5 (solid green circles), and 11 (solid red circles). The surface tension of the single-surfactant solution at pH 7 is also shown (open black circles). 3 repeated measurements were performed and their average values are shown. Error bars represent the 95% confidence intervals.

At a given surfactant concentration in the presence of gelatin, the surface tension decreased with decreasing pH in the solution phase. Notably, the stepwise decrease in surface tension was observed even at pH 9.5 (isoelectric point of gelatin) and 11. These results suggest two points: (i) complex formation between the surfactant and gelatin molecules is driven not only by electrostatic interactions but also by hydrophobic interactions,^30^ and (ii) the adsorbed film is more closely packed at pH 7 than at higher pH, likely due to electrostatic attraction between the negatively charged surfactant and the positively charged gelatin molecules. The attractive electrostatic interaction leads to charge neutralization, which increases the hydrophobicity of gelatin molecules. Consequently, the surfactant–gelatin complex is reasonably expected to adsorb to the air/solution interface even at relatively low surfactant concentrations. The surfactant–gelatin complex is gradually recharged with increasing surfactant concentration. This results from further adsorption of surfactant molecules to the complex mainly due to hydrophobic interactions. Hence the complex adsorbed at the air/solution interface moves into a liquid phase, and instead, the surfactant monolayer is formed at high surfactant concentrations.

When oppositely charged surfactants and polyelectrolytes are mixed in aqueous media, phase separation sometimes occurs. “Coacervate” is a polyelectrolyte-rich liquid phase separated from a polyelectrolyte-poor liquid phase (continuous phase). In our system, solutions in the intermediate surfactant concentration region were visually turbid at pH 7, although no precipitation was observed during the surface tension measurements. This observation suggests that the surfactant–gelatin complex forms coacervates dispersed in the aqueous media. This was supported by optical microscopy, as shown in Figure S2. It has been reported that the surface tension decreases in two stages with respect to surfactant concentration even in the presence of coacervates.^31–33^ Increasing the pH reduced turbidity, and visually transparent solutions were obtained at pH 11.

### Foam Stability

We examined the foamability and stability of foams by vigorously hand-shaking the prepared samples. Figure 2 shows the visual observations for the single-surfactant and mixed surfactant–gelatin systems at different surfactant concentrations. The gelatin concentration was fixed at 0.5 mass %, and the surfactant concentration was varied. The solution pH was approximately 7. Foamability increased with increasing surfactant concentration, a trend observed in both the single-surfactant and mixed surfactant–gelatin systems. In addition, the mixed surfactant–gelatin system exhibited higher foamability than the single-surfactant system at a given surfactant concentration (<12 mmol/dm^3^).

**Figure 2:**
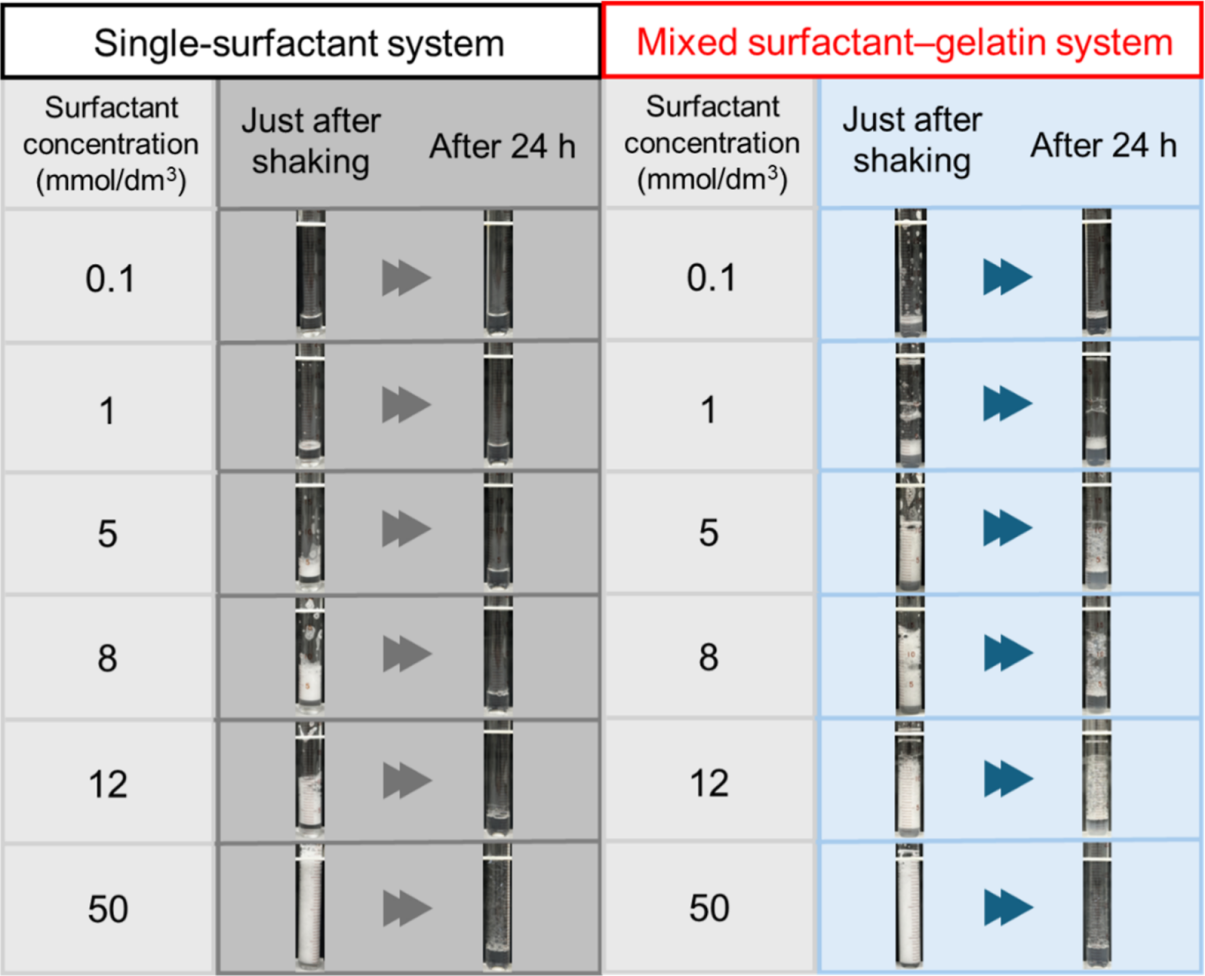
Visual observations of foams generated by vigorous hand-shaking 20× with and without gelatin (0.5 mass %). The surfactant concentration was varied from 0.1 to 50 mmol/dm^3^, and the solution pH was approximately 7. Images were taken immediately after shaking and 24 h after shaking.

The stability of foams was also evaluated based on the visual observations shown in Figures 2 (pH 7) and S3 (pH 9.5 and 11). Foam stability was defined as the ratio of the foam height measured 24 h after hand-shaking to that measured immediately after hand-shaking. The results are summarized in Figure 3. At pH 7, the addition of gelatin to the surfactant solution improved foam stability (<12 mmol/dm^3^), although dry foams still remained at 50 mmol/dm^3^ in the single-surfactant system. More importantly, the highest stability was observed at a surfactant concentration of 5 mmol/dm^3^ in the presence of gelatin. These findings suggest that the adsorbed film composed of the anionic surfactant and gelatin contributes to the increased foam stability. This is also supported by the experimental finding that the lower foam stability was observed at the surfactant concentration of 50 mmol/dm^3^ in the presence of gelatin, where the surfactant monolayer is expected to be formed at the air/solution interface. The observed changes in foam stability will be discussed in relation to the interfacial shear rheological data.

**Figure 3:**
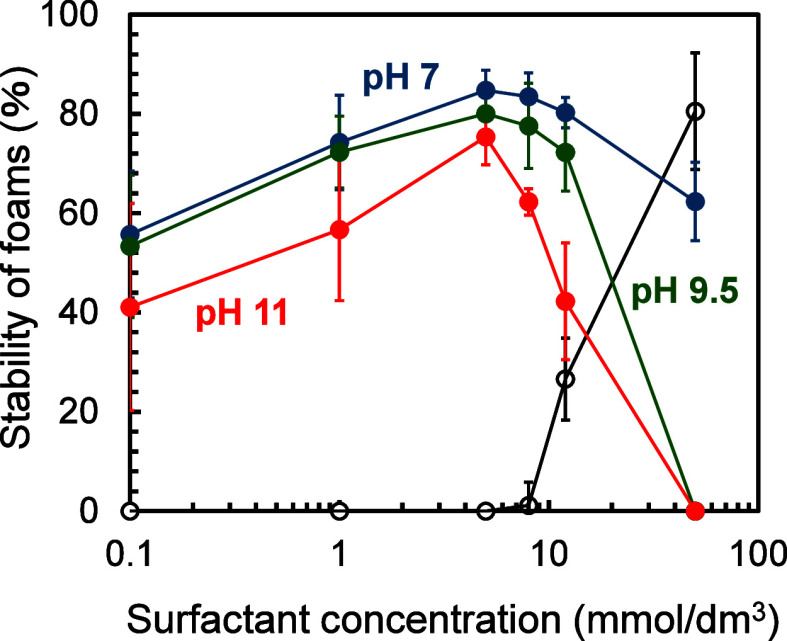
Stability of foams evaluated from the visual observations. The solution pH was adjusted to approximately 7 (solid blue circles), 9.5 (solid green circles), and 11 (solid red circles). The stability of foams for the single-surfactant solution at pH 7 is also shown (open black circles). Three repeated measurements were performed and their average values are shown. Error bars represent the 95% confidence intervals.

The stability of foams was further examined at pH 7 using the dynamic foam analyzer. Figure 4 shows the foam morphology in the presence of gelatin at surfactant concentrations of 1, 5, and 50 mmol/dm^3^. As described in the Materials and Methods section, these morphologies were monitored at a height of approximately 7 cm from the bottom of the cylinder. This height is slightly above the liquid phase, although the volume of the liquid phase gradually increased over time due to defoaming. At surfactant concentrations of 1 and 5 mmol/dm^3^, closely packed small bubbles were maintained even after 60 min. These small bubbles constitute wet foams and appear to resist drainage and coalescence. This suggests that drainage of the liquid film and bubble coalescence are key mechanisms leading to foam destabilization. Electrostatic repulsions between two opposing air/liquid interfaces may also increase disjoining pressure and thereby suppress drainage. It seems likely, however, that the impact of this effect is limited at the surfactant concentration of 5 mmol/dm^3^, where the complex adsorbed at the air/liquid interface is expected to be neutralized significantly and therefore hydrophobic. In contrast, at 50 mmol/dm^3^, loosely packed large bubbles with polygonal shapes were observed, indicating that drainage from the liquid film progressed and bubble coalescence occurred under the experimental conditions.

**Figure 4:**
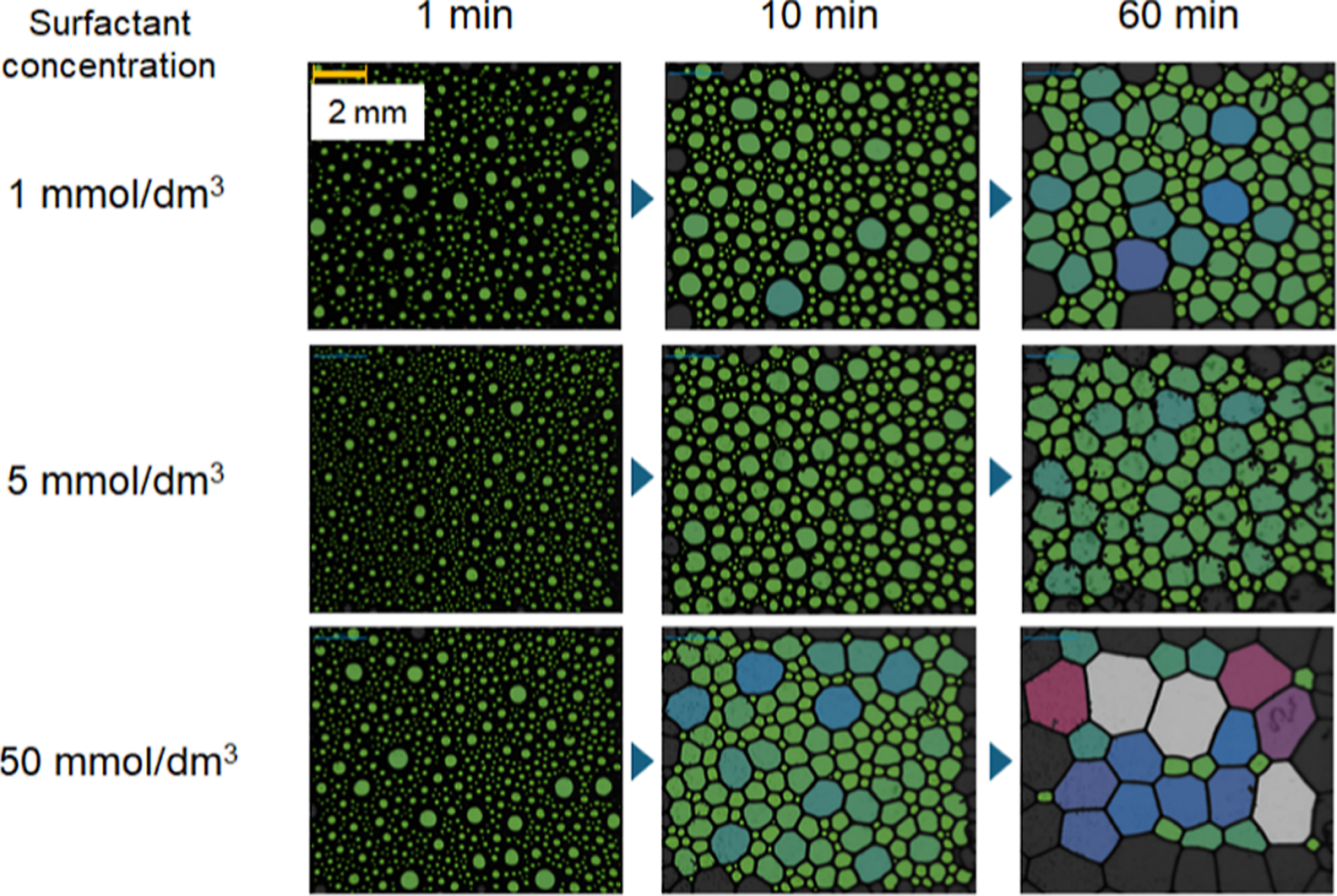
Foam morphology observed using the dynamic foam analyzer. The surfactant concentrations were set at 1, 5, and 50 mmol/dm^3^, and the gelatin concentration was fixed at 0.5 mass %. The solution pH was approximately 7. Images were recorded at 1, 10, and 60 min after bubbling was stopped. Size distributions of foams observed in each image are presented in Figure S4.

In Figure 3, the maximum foam stability was observed at the surfactant concentration of 5 mmol/dm^3^ in the presence of gelatin, regardless of pH. Notably, foam stability increased as pH decreased, corresponding to the lower surface tension measured at pH 7 compared with pH 9.5 and 11 (Figure 1). Interestingly, even at pH 11, where both the anionic surfactant and gelatin carry net negative charges, the addition of gelatin still enhanced foam stability. Hereafter, we discuss foam stability and interfacial shear rheology as functions of surfactant concentration (at a fixed pH of 7) and pH.

### Interfacial Shear Rheology

We assessed the interfacial rheological properties of the adsorbed film using a rheometer equipped with the bicone-type geometry. Frequency-sweep measurements were performed under a constant strain of 1%. This strain was assumed to be in the linear viscoelastic region based on strain-sweep measurements (Figure S5). Additionally, these frequency-sweep measurements were performed at least 15 min after the instrument was set up. Dynamic rheological measurements indicated that stable shear moduli (*G*′ and *G*″) were achieved during the equilibration period (Figure S6).

Figure 5 shows the results obtained for the mixed surfactant–gelatin system at pH 7. The gelatin concentration was fixed at 0.5 mass %, and the surfactant concentration was varied at (a) 1, (b) 5, (c) 8, and (d) 12 mmol/dm^3^. *G*′ and *G*″ were plotted as functions of angular frequency. No significant *G*′ data were obtained in the single-surfactant system, indicating that the interfacial phase behaves as a purely viscous fluid. Similarly, *G*′ data were scattered in the mixed surfactant–gelatin system at 1 mmol/dm^3^, likely due to insufficient torque, suggesting a weak mechanical response from the interface, as seen in Figure 5a. In contrast, significant *G*′ and *G*″ values were obtained at surfactant concentrations of 5, 8, and 12 mmol/dm^3^ in the presence of gelatin. At these concentrations, the surface tension results (Figure 1) suggested the formation of surfactant–gelatin complex adsorbed at the air/solution interface. Across nearly the entire frequency range investigated, *G*′ exceeded *G*″ at a given frequency, indicating that the mixed film is elasticity-dominant.

**Figure 5:**
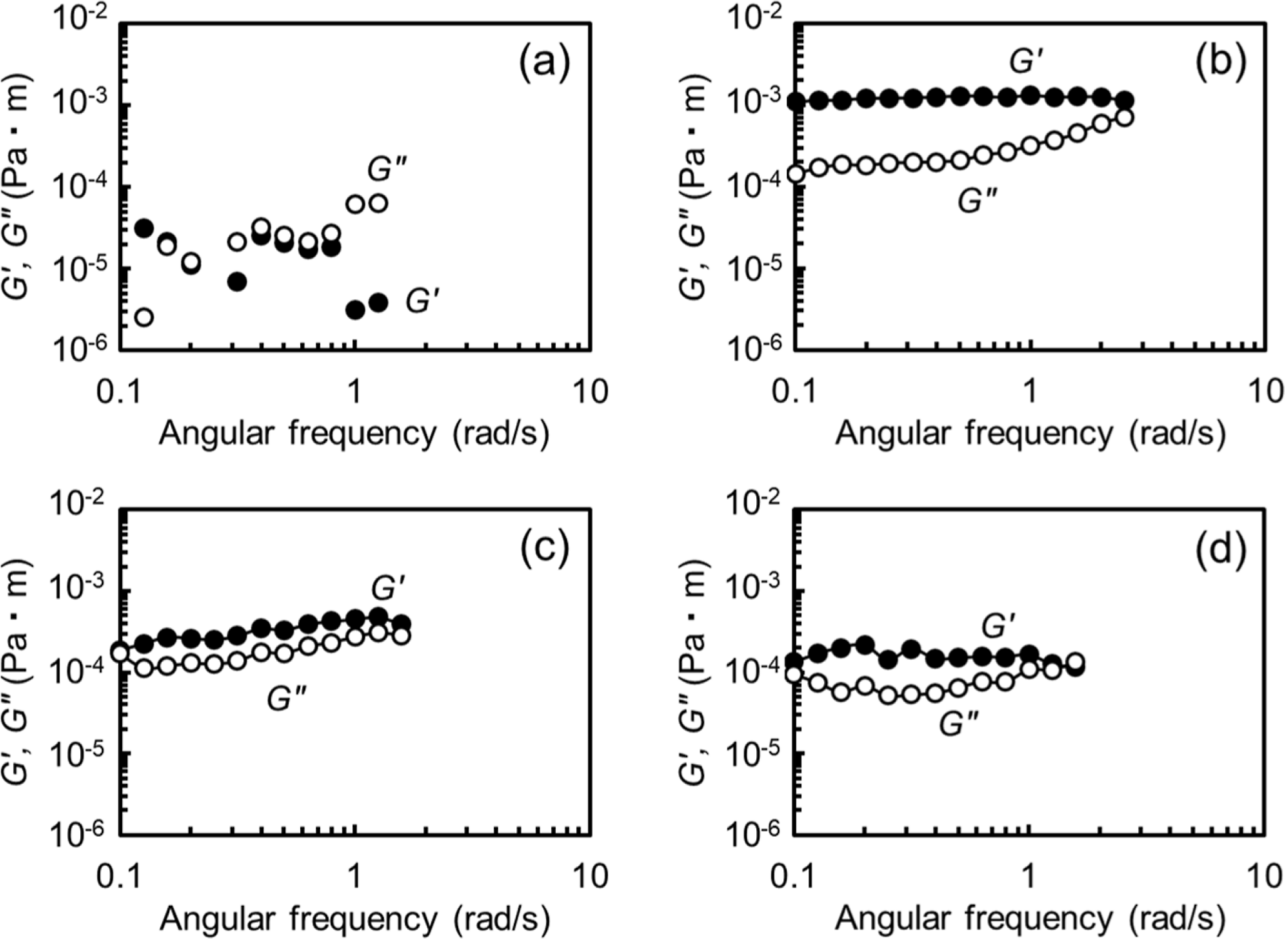
Interfacial shear storage modulus (*G*′) and loss modulus (*G*″) as functions of angular frequency in the presence of gelatin (0.5 mass %). The surfactant concentrations were set at (a) 1, (b) 5, (c) 8, and (d) 12 mmol/dm^3^. The solution pH was approximately 7.

The *G*′ and *G*″ data obtained at an angular frequency of 0.5 rad/s were plotted in Figure 6 as functions of surfactant concentration. The loss tangent (tan δ = *G*″/*G*′) is also shown. The maximum *G*′ and *G*″ values and the minimum tan δ value were observed at a surfactant concentration of 5 mmol/dm^3^ in the presence of gelatin. The corresponding three-dimensional bulk *G*′ and *G*″ data were shown in Figure S7, where a gradual increase in *G*′ was observed with increasing surfactant concentration instead of the local maximum at 5 mmol/dm^3^. These findings suggest that the mixed film is elasticity-dominant, reducing the likelihood of bubble coalescence. The increased interfacial *G*′ and *G*″ values at the surfactant concentration of 5 mmol/dm^3^ are also expected to suppress drainage from the liquid film. Consequently, foam stability was highest at this surfactant concentration.

**Figure 6:**
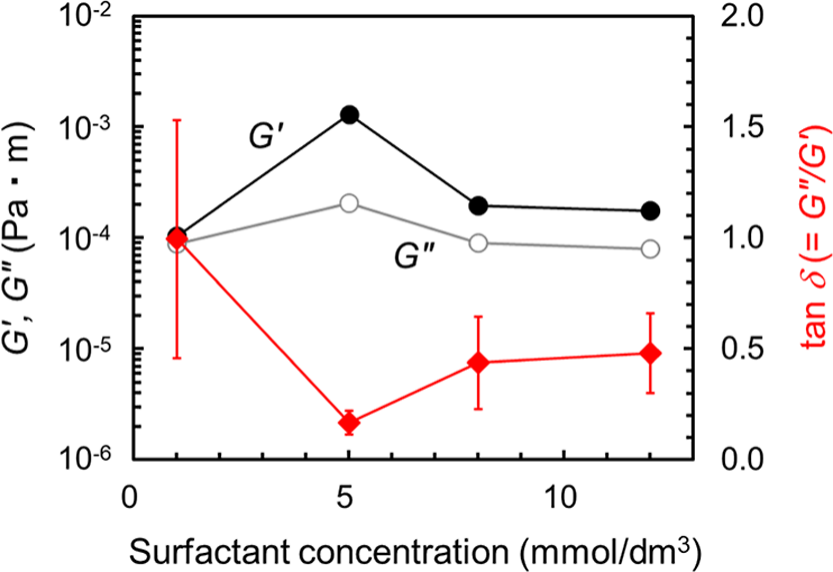
Interfacial shear storage modulus (*G*′), loss modulus (*G*″), and loss tangent (tan δ = *G*″/*G*′) as functions of surfactant concentration in the presence of gelatin (0.5 mass %). The data obtained at the angular frequency of 0.5 rad/s are plotted. Three repeated measurements were performed and their average values are shown. The 95% confidence intervals for tan δ are shown in the figure.

The interfacial stress-controlled rheometry using a Teflon-coated magnetic needle revealed that the interfacial shear moduli (*G*′ and *G*″) reached their maximum values at an equivalent mixing molar ratio of a cationic surfactant and an anionic polyelectrolyte in monomer units.^34,35^ This behavior is attributed to the “gelation” of the oppositely charged surfactant and polyelectrolyte, which produces an elasticity-dominant viscoelastic film at the air/solution interface. Such interfacial gelation markedly retards drainage from thin liquid films and suppresses bubble coalescence, thereby enhancing foam stability at this mixing molar ratio.^34,35^ Although the charge density of gelatin (and therefore the precise mixing molar ratio in monomer units) is unknown in our system, the similarity in surface tension behavior suggests that analogous interfacial gelation occurs; that is, interfacial gelation arises under conditions in which the surfactant–polymer complex exhibits surface activity.^34,35^

As mentioned earlier, the surfactant–gelatin complex tends to form coacervates at lower pH. In our case, the influence of these coacervates, which are dispersed in the aqueous phase, on foam stability is still uncertain. A plausible hypothesis is that the presence of coacervates in the liquid films and/or Plateau borders slows drainage and thereby contributes to improved foam stability. Evidence supporting this scenario can be found in previous studies,^36–39^ although these studies employed complex coacervates or heat-denatured colloidal particles composed of two polymers rather than surfactants. Notably, one of these studies suggested that complexes (or coacervates) form around bubble surfaces, producing foams with relatively high viscoelasticity.^37^ This mechanism is consistent with our observations. In our system, interfacial gelation occurs, producing the elasticity-dominant interfacial film that retards drainage from thin liquid films and suppresses bubble coalescence. Thus, while the chemical nature of the complexes differs, the stabilization mechanism—complex formation leading to slower drainage and higher resistance against coalescence—appears to be common across these systems.

Figure 7 shows the *G*′ and *G*″ values for the surfactant–gelatin mixtures at different pH values (7, 9.5, and 11). These values were obtained from frequency-sweep measurements and extracted at an angular frequency of 0.5 rad/s. The surfactant and gelatin concentrations were set at 5 mmol/dm^3^ and 0.5 mass %, respectively, where the highest foam stability was observed under each pH condition. The loss tangent (tan δ) is also shown in this figure. The corresponding three-dimensional bulk *G*′, *G*″, and tan δ data were shown in Figure S8. At pH 7 and 9.5, the two-dimensional interfacial tan δ value was less than 1 (i.e., *G*″ < *G*′), indicating that an elasticity-dominant, gel-like film formed at the air/solution interface. Comparison of the rheological data across pH conditions shows that decreasing pH resulted in increased *G*′ and *G*″ and decreased tan δ. These results indicate a stronger rheological response at low pH, arising from the formation of a more elastic interfacial film. This behavior is attributed to interfacial gelation between the oppositely charged surfactant and gelatin at pH 7, as discussed earlier.

**Figure 7:**
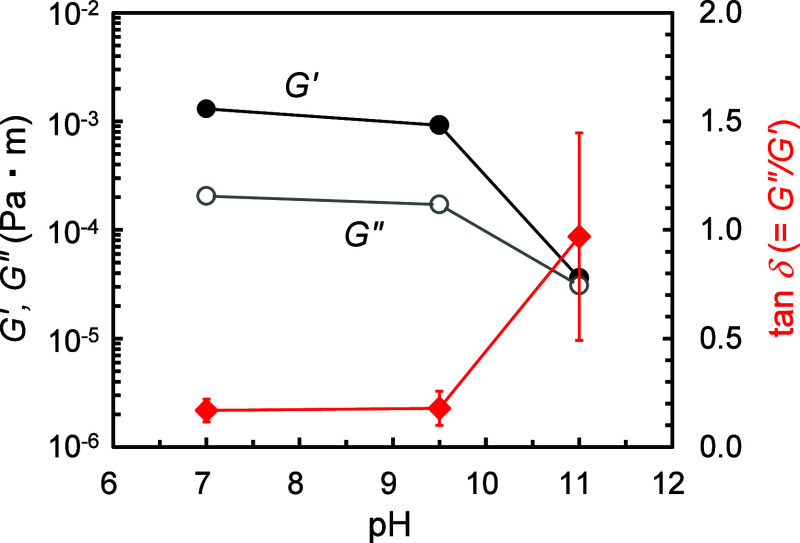
Interfacial shear storage modulus (*G*′), loss modulus (*G*″), and loss tangent (tan δ = *G*″/*G*′) in the presence of gelatin (0.5 mass %) at different pH values. The surfactant concentration was fixed at 5 mmol/dm^3^. Frequency-sweep measurements were performed under a constant strain of 1%, and the data extracted at the angular frequency of 0.5 rad/s are plotted. Three repeated measurements were performed and their average values are shown. The 95% confidence intervals for tan δ are shown in the figure.

## Conclusions

Interfacial shear rheological measurements using the bicone-type geometry revealed that the mixture of the anionic amino acid-based surfactant and the zwitterionic polyelectrolyte (gelatin) forms a viscoelastic film adsorbed at the air/solution interface. At a surfactant concentration of 5 mmol/dm^3^ in the presence of gelatin (0.5 mass %) at pH 7, the interfacial shear moduli (*G*′ and *G*″) obtained from frequency-sweep measurements reached their maximum values, and the loss tangent (tan δ) was minimized, indicating the formation of an elasticity-dominant, gel-like film. In other words, interfacial gelation occurred between the oppositely charged components. Such gelation enhances foam stability by suppressing drainage from the liquid film and bubble coalescence. Furthermore, the interfacial rheological measurements confirmed that lower pH promotes stronger elastic responses, consistent with the lower surface tension and higher foam stability observed at pH 7. These findings support the hypothesis that interfacial gelation plays a key role in stabilizing foams in the surfactant–gelatin system.

Interfacial shear rheology enables the measurement of rheological properties in cloudy or dispersed samples, which can be difficult to analyze using interfacial dilational techniques. Moreover, interfacial shear rheology is expected to allow measurements for samples in which the surfactant concentration is high and the adsorption rate exceeds the interfacial response, as well as for samples in which a relatively thick film forms at the interface. Interfacial shear rheology using the bicone-type geometry is therefore anticipated to provide a practical approach for predicting the long-term stability of foams in various industrial applications.

## Supplementary Material



## Notes

Figure S1. Zeta potentials of acid-treated gelatin as a function of pH. Figure S2. Optical microscope image of coacervates dispersed in aqueous media. The surfactant and gelatin concentrations were set at 5 mmol/dm^3^ and 0.5 mass %, respectively. The solution pH was approximately 7. Figure S3. Visual observations of foams generated by vigorous hand-shaking 20× with and without gelatin (0.5 mass %). The surfactant concentration was varied from 0.1 to 50 mmol/dm^3^ and the solution pH was adjusted to 9.5 and 11. Figure S4. Size distribution of foams observed in Figure 4. Figure S5. Interfacial shear storage modulus (*G*′) and loss modulus (*G*″) as functions of strain in the presence of gelatin (0.5 mass %). The surfactant concentration was set at 5 mmol/dm^3^, and the solution pH was approximately 7. Figure S6. Shear storage modulus (*G*′) and loss modulus (*G*″) as functions of time in the presence of gelatin (0.5 mass %). The surfactant concentration was set at 5 mmol/dm^3^, and the solution pH was approximately 7. Figure S7. Three-dimensional bulk shear storage modulus (*G*′), loss modulus (*G*″), and loss tangent (tan δ = *G*″/*G*′) as functions of surfactant concentration in the presence of gelatin (0.5 mass %). Figure S8. Three-dimensional bulk shear storage modulus (*G*′), loss modulus (*G*″), and loss tangent (tan δ = *G*″/*G*′) in the presence of gelatin (0.5 mass %) at different pH values (PDF)
