# Supplementary material for: Foam Stability in Aqueous Systems Containing an Amino Acid-Based Surfactant and Gelatin: An Interfacial Shear Rheology Perspective
Source: Langmuir. 2026 Apr 10;42(16):10888–95. doi: 10.1021/acs.langmuir.5c05853 (PMC13130969; doi:10.1021/acs.langmuir.5c05853)
Supplement: Supplementary file 1 [file la5c05853_si_001.pdf]

Supporting Information:

"Foam Stability in Aqueous Systems Containing an Amino Acid-Based Surfactant and Gelatin: An Interfacial Shear Rheology Perspective"

Kenta Asai,[1] Kyosuke Arakawa,[1] Koji Tsuchiya,[2] Shiho Yada,[3] Yukishige Kondo,[2,3] Yoshifumi Yamagata,[2,4] Hideki Sakai,[1,2] and Kenichi Sakai[1,2*]

Corresponding author e-mail: k-sakai@rs.tus.ac.jp

1 Department of Pure and Applied Chemistry, Faculty of Science and Technology, Tokyo University of Science, 2641 Yamazaki, Noda, Chiba 278-8510, Japan.

2 Research Institute for Science and Technology, Tokyo University of Science, 2641 Yamazaki, Noda, Chiba 278-8510, Japan.

3 Department of Industrial Chemistry, Faculty of Engineering, Tokyo University of Science, 6-3-1 Niijyuku, Katsushika, Tokyo 125-8585, Japan.

4 Anton Paar Japan K.K. Riverside Sumida 1F, 1-19-9 Tsutsumi-dori, Sumida, Tokyo 131-0034, Japan.

Number of pages: 6

Number of figures: 8

Number of schemes: 0

Number of tables: 0

Table of Contents in Supporting Information

1. Results

1. Results

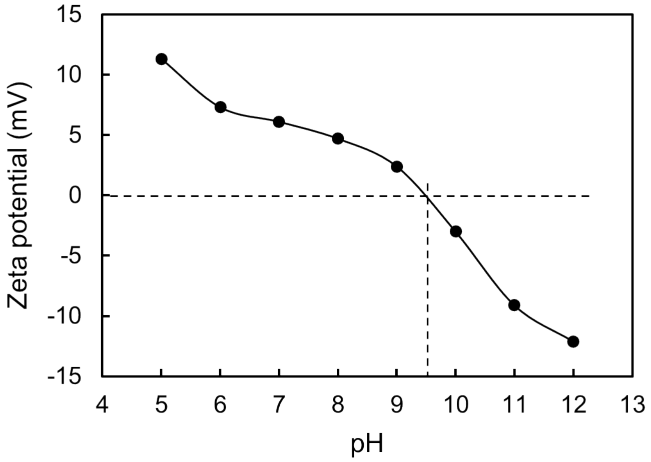

Figure S1. Zeta potentials of acid-treated gelatin (0.5 mass%) as a function of pH. These measurements were performed using an Anton Paar Litesizer 500 system.

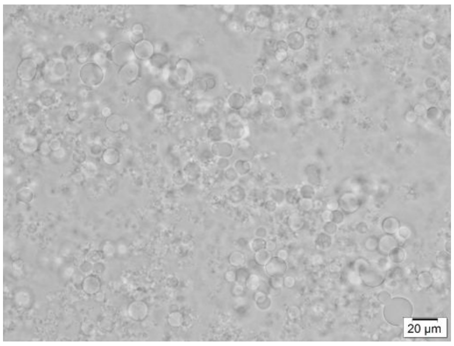

Figure S2. Optical microscope image of coacervates dispersed in aqueous media. The surfactant and gelatin concentrations were set at 5 mmol/dm^3 and 0.5 mass%, respectively. The solution pH was approximately 7. This observation was performed using an Olympus IX73 inverted microscope system.

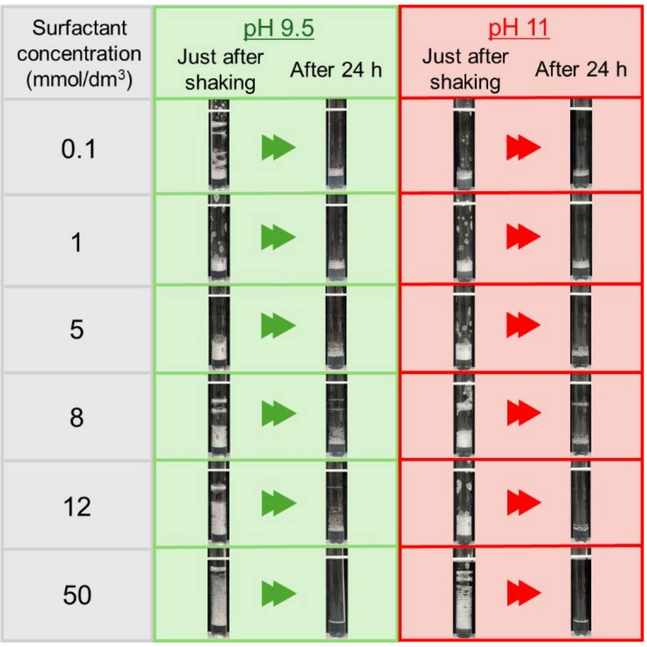

Figure S3. Visual observations of foams generated by vigorous hand-shaking twenty times with and without gelatin (0.5 mass%). The surfactant concentration was varied from 0.1 to 50 mmol/dm^3, and the solution pH was adjusted to 9.5 and 11. Images were taken immediately after shaking and 24 h after shaking.

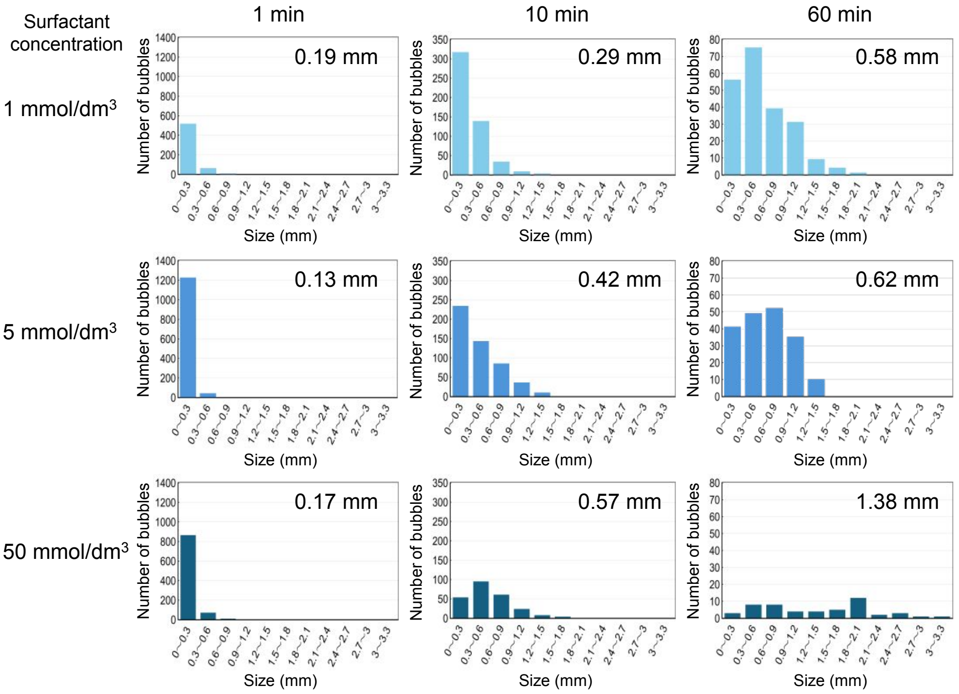

Figure S4. Size distribution of foams observed in Figure 4. Foam sizes were analyzed using ImageJ. The measured area was approximated as that of a circle, and the corresponding diameter is shown. The average foam size for each image is indicated in each histogram.

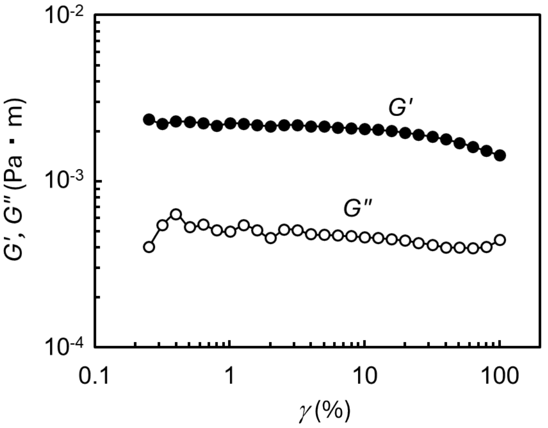

Figure S5. Interfacial shear storage modulus (G') and loss modulus (G'') as functions of strain in the presence of gelatin (0.5 mass%). The surfactant concentration was set at 5 mmol/dm^3, and the solution pH was approximately 7. These measurements were performed under a constant angular frequency of 1 rad/s.

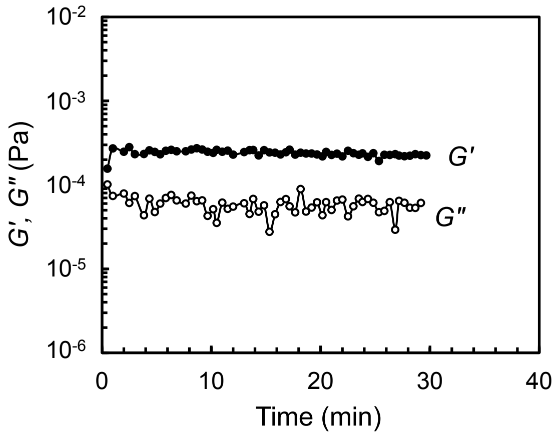

Figure S6. Shear storage modulus (G') and loss modulus (G'') as functions of time in the presence of gelatin (0.5 mass%). The surfactant concentration was set at 5 mmol/dm^3, and the solution pH was approximately 7. These measurements were performed under a constant strain of 1% and a constant angular frequency of 0.5 rad/s. The bicone-type geometry was used for these measurements, but subsequent analyses for evaluating interfacial G' and G'' were not applied in these results.

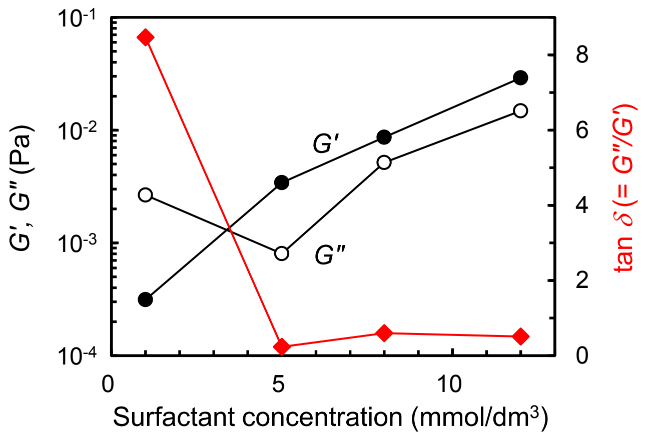

Figure S7. Three-dimensional bulk shear storage modulus (G'), loss modulus (G''), and loss tangent ($\tan\delta = G''/G'$) as functions of surfactant concentration in the presence of gelatin (0.5 mass%). The solution pH was approximately 7. The data obtained at the angular frequency of 0.5 rad/s are plotted under a constant strain of 1%.

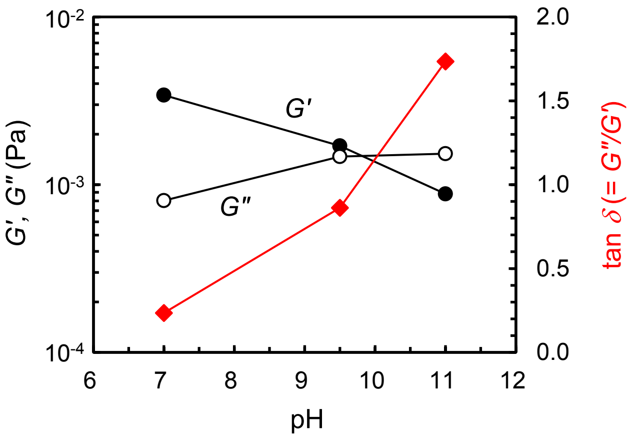

Figure S8. Three-dimensional bulk shear storage modulus (*G*′), loss modulus (*G*″), and loss tangent (tan δ = G''/G') in the presence of gelatin (0.5 mass%) at different pH values. The surfactant concentration was fixed at 5 mmol/dm^3. Frequency-sweep measurements were performed under a constant strain of 1%, and the data extracted at the angular frequency of 0.5 rad/s are plotted.
